# Proteomic Profiling and Rhizosphere-Associated Microbial Communities Reveal Adaptive Mechanisms of *Dioclea apurensis* Kunth in Eastern Amazon’s Rehabilitating Minelands

**DOI:** 10.3390/plants11050712

**Published:** 2022-03-07

**Authors:** Sidney Vasconcelos do Nascimento, Paulo Henrique de Oliveira Costa, Hector Herrera, Cecílio Frois Caldeira, Markus Gastauer, Silvio Junio Ramos, Guilherme Oliveira, Rafael Borges da Silva Valadares

**Affiliations:** 1 Instituto Tecnológico Vale, Rua Boaventura da Silva 955, Belém CEP 66050-090, Brazil; sidney.nascimento@pq.itv.org (S.V.d.N.); paulo.henrique.costa@pq.itv.org (P.H.d.O.C.); cecilio.caldeira@itv.org (C.F.C.); markus.gastauer@itv.org (M.G.); silvio.ramos@itv.org (S.J.R.); guilherme.oliveira@itv.org (G.O.); 2 Programa de Pós-Graduacão em Genética e Biologia Molecular, Universidade Federal do Pará, Belém CEP 66075-110, Brazil; 3 Departamento de Ciencias Forestales, Universidad de La Frontera, Temuco 4811230, Chile; hector.herrera@ufrontera.cl

**Keywords:** abiotic stress, proteomic, rehabilitating minelands, rhizosphere, symbiosis

## Abstract

*Dioclea apurensis* Kunth is native to ferruginous rocky outcrops (known as *canga*) in the eastern Amazon. Native *cangas* are considered hotspots of biological diversity and have one of the largest iron ore deposits in the world. There, *D. apurensis* can grow in post-mining areas where molecular mechanisms and rhizospheric interactions with soil microorganisms are expected to contribute to their establishment in rehabilitating minelands (RM). In this study, we compare the root proteomic profile and rhizosphere-associated bacterial and fungal communities of *D. apurensis* growing in *canga* and RM to characterize the main mechanisms that allow the growth and establishment in post-mining areas. The results showed that proteins involved in response to oxidative stress, drought, excess of iron, and phosphorus deficiency showed higher levels in *canga* and, therefore, helped explain its high establishment rates in RM. Rhizospheric selectivity of microorganisms was more evident in *canga.* The microbial community structure was mostly different between the two habitats, denoting that despite having its preferences, *D. apurensis* can associate with beneficial soil microorganisms without specificity. Therefore, its good performance in RM can also be improved or attributed to its ability to cope with beneficial soil-borne microorganisms. Native plants with such adaptations must be used to enhance the rehabilitation process.

## 1. Introduction

Iron ore extraction in the eastern Amazon occurs mainly in ferruginous mountain outcrops surrounded by rainforests in the Carajás mineral province, covering some of the largest iron reservoirs of the world [1]. This ecosystem is characterized by a high plant diversity that forms different phytophysiognomies known as *canga* [2]. The plant communities that grow in *canga* are subjected to adverse environmental conditions such as intense ultraviolet (UV) radiation, high temperatures (soil and air), which, combined with constant winds, increase evapotranspiration processes [3]. At the same time, water acquisition is hampered by poor water-holding capacity influenced by soil composition and shallow soil formation, which tends to intensify the effects of water deficit [2,3]. Additionally, the oxidic soils from *canga* are characterized by low availability of soluble phosphorus (P) caused by high adsorption of P to iron and aluminum oxides [4]. Plants require several adaptations to grow and colonize such ecosystems. A robust adaptive metabolism and symbiotic associations with soil microorganisms can lead to a successful establishment under these harsh environmental conditions.

Iron mining in Brazil occurs essentially in open-cast mines, including those in the Carajás mineral province [5]. The areas of iron ore extraction are characterized by the removal of vegetation and soil, and steep slopes [6]. Recent studies have evaluated different plants in the rehabilitation of areas affected by iron mining in the Amazon, including the reintroduction of native species [7]. These species have a crucial role in the success of mineland rehabilitation, reducing local biodiversity loss and contributing to the progressive recovery of ecological services associated with *canga*. Considering that revegetation is a fundamental technique for the rehabilitation of minelands, there is a need to identify native species able to cope with the stressful environmental conditions of post-mining areas in *canga* and understand the adaptive mechanisms used by such plants [7,8,9].

Plants from the genus *Dioclea* (Fabaceae) can grow in nearby mining areas in *canga* [10]. Considered a metallophyte species, *Dioclea apurensis* Kunth was listed as one of the most promising plants for mineland rehabilitation [11]. *D. apurensis* has been frequently used for the revegetation of waste piles and open-cast mines in rehabilitating minelands (RM) projects due to the ease of finding in native *canga* ecosystems, high seed production, and rapid growth [12]. However, the molecular mechanisms underlining the establishment of this species in RM have been scarcely explored.

Symbiosis with soil-borne microorganisms is a crucial step to promote the plant colonization of post-mining areas [13,14]. In the rhizosphere (soil section in contact with the plant roots), several microbial taxa are influenced by the plant metabolism, many of which have plant growth promoting traits that are useful to improve the plant establishment and growth under harmful ecosystems such as RM [15,16]. The mechanism by which the rhizosphere-associated microbial communities support the plant growth include production of plant growth regulators, improved water acquisition, direct nutrient solubilization, protection against phytopathogens, promotion of abiotic stress tolerance, among others [17,18]. Therefore, understanding how native plants interact with microorganisms inhabiting the soil substrate is key to characterize the beneficial symbiosis in rehabilitating Amazonian *cangas*.

Despite recognizing the importance of reintroducing native species for mineland rehabilitation, few studies analyze the protein profile of native plants used in rehabilitation programs and the interaction with microbiological communities in plants growing in post-mining areas. The aims of this study were: (i) to analyze the protein profile of *D. apurensis* roots from plants growing in RM; and (ii) to identify rhizosphere microorganisms that contribute to the adaptation of *D. apurensis* in RM. For this, we compare the protein profiles and rhizosphere-associated microorganisms from plants growing in *canga* (their natural distribution habitat) and from plants growing in the RM.

## 2. Results

### 2.1. Physical and Chemical Properties of Canga and Mining Area Soil Substrates

The soil substrates of RM showed a greater proportion of clay, while *canga* soils showed higher proportions of sand (Table 1). The soils from *canga* were more acidic than RM soils (Table 1). *Canga* soils showed more organic matter content than soils from RM (Table 1). Total nitrogen (N) was higher in *canga* soils, whereas available P was higher in RM soil substrates (Table 1). The concentrations of metals differs between environments; copper and iron were higher in *canga* soils, whereas manganese and zinc were higher in RM soils (Table 1).

### 2.2. Protein Profiles, Annotation, and Functional Enrichment

**Figure 1:**
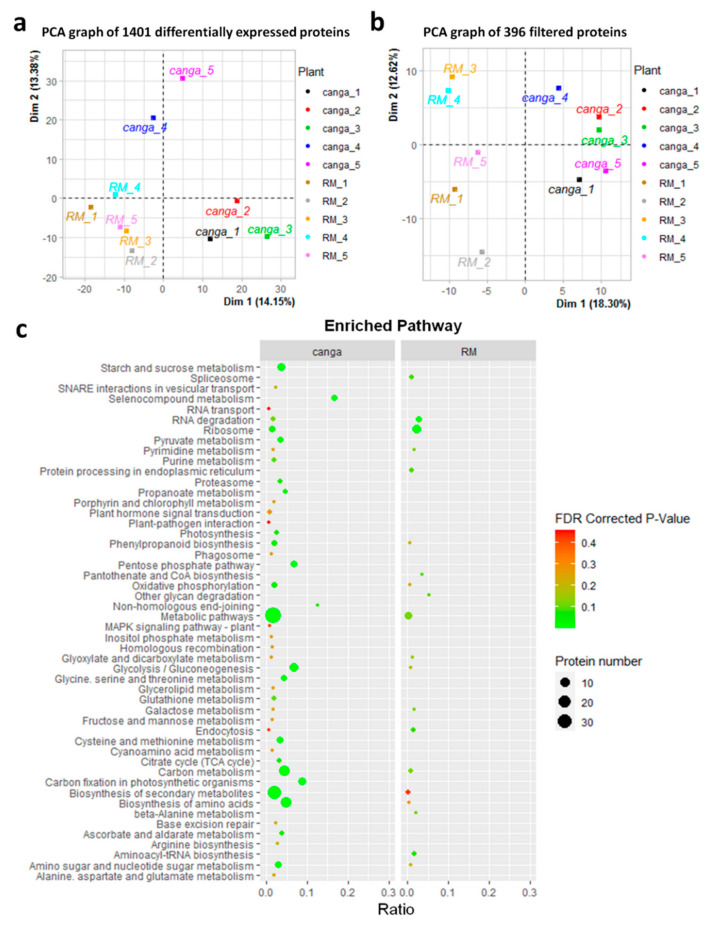
PCA and enrichment analysis of the proteins with differential abundances in *D. apurensis* roots sampled from *canga* (*canga*) or in rehabilitating mineland (RM). (**a**) PCA of 1401 differentially accumulated proteins comparing five plants from *canga* with five from RM. (**b**) PCA of differentially accumulated and filtered proteins based on *p* < 0.05 and fold change ≥ 1.5. (**c**) Enrichment analysis of the most accumulated proteins in different biological processes. The significantly enriched KEGG pathways were indicated as dots (FDR corrected *p* < 0.05, Fisher’s exact test, Benjamini and Hochberg FDR correction method). The dot sizes represent the number of proteins included in each pathway. The x-axis represents the ratio of the number of differentially accumulated proteins and the number of all proteins in the pathway.

A total of 1401 proteins were successfully identified and quantified, with 396 showing significant differential abundance (*p* < 0.05) between the roots from both environments (Dataset S1), being more abundant in roots from *canga*. Only two proteins were exclusively detected in the roots from RM, while 19 were identified only in the roots from *canga* (Dataset S1). Within the total proteins identified, 291 showed higher levels in roots from *canga*, while 105 were showed higher levels in roots from RM. The principal component analysis (PCA) of the 1401 proteins (Figure 1a) and 396 filtered proteins (Figure 1b) showed the separation between root samples from *canga* and roots from RM.

The enrichment analysis showed that the most accumulated proteins in *canga* act in 44 main processes, while the most accumulated proteins in RM participate in at least 20 processes (Figure 1c). In the roots from both sampling sites, the most prominent categories included proteins involved in response to stimuli, mainly related to abiotic stresses, and proteins involved in growth and reproduction, including proteins involved in carbon metabolism and biosynthesis of secondary metabolites (Figure 1c). Proteins involved in these pathways, especially in secondary metabolite biosynthesis, including amino acid biosynthesis, and carbon metabolism, were more accumulated in *canga*. Figure S1a,b show the 40 main biological processes from the functional annotation of the most accumulated proteins in the roots from *canga* and RM, respectively.

### 2.3. Protein–Protein Interactions (PPI)

**Figure 2:**
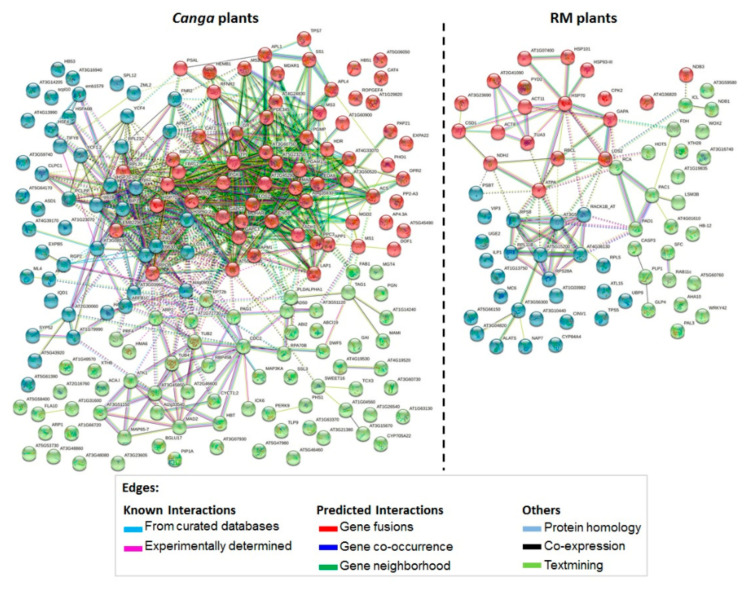
Protein–protein interaction network of the proteins with differential abundances in *D. apurensis* in *canga* (to the left) and RM (to the right) separated into 3 *K*-means clusters illustrated in different colors. The proteins used for analysis are presented in Dataset S2. Gene symbols were obtained using homologous proteins from the *Arabidopsis thaliana* database from uniprot.org, accessed on 10 October 2021. Edges represent protein–protein interactions as described in the figure. Number of edges between in *canga* plants: 697. Number of edges between RM plants: 54. Dotted lines represent edges between clusters. PPI enrichment *p*-value in *canga* plants: 1 × 10^−16^. PPI enrichment in RM plants: <1.4 × 10^−6^.

The analysis of PPI showed 178 proteins with higher levels in the roots from *canga* (Figure 2, Dataset S2) and 69 proteins in the roots from RM (Figure 2, Dataset S2). The enrichment and PPI analysis showed an essential co-occurrence of a large part of the proteins accumulated in plants of each environment. These proteins were grouped into three highly interacting protein clusters, with a PPI enrichment *p*-value of 1 × 10^−16^ in *canga* plants and *p*-value < 1.4 × 10^−6^ in RM plants (Figure 2, Dataset S2).

In *canga* plants, the functional enrichment analysis showed that the cluster in red includes 63 proteins mainly involved in energy metabolism and secondary metabolite biosynthesis, including amino acid biosynthesis. The green cluster comprised 69 proteins related to the cell cycle and response, especially abiotic stresses such as osmotic stress. The blue cluster contains 46 proteins, mainly including binding proteins such as nucleic acid binding proteins, structural components of ribosomes, and protein folding. In RM plants, the red cluster was composed of 19 proteins related to abiotic stimuli, especially temperature. The cluster in green contains 24 proteins categorized by the functional enrichment analysis of the term “cellular anatomical entity”, which includes proteins involved in carbon metabolism and biosynthesis of secondary metabolites. The 26 proteins included in the blue cluster of plants on RM were categorized mainly into cellular processes such as gene expression and protein metabolic process (Figure 2, Dataset S2).

### 2.4. Microbial Diversity

**Figure 3:**
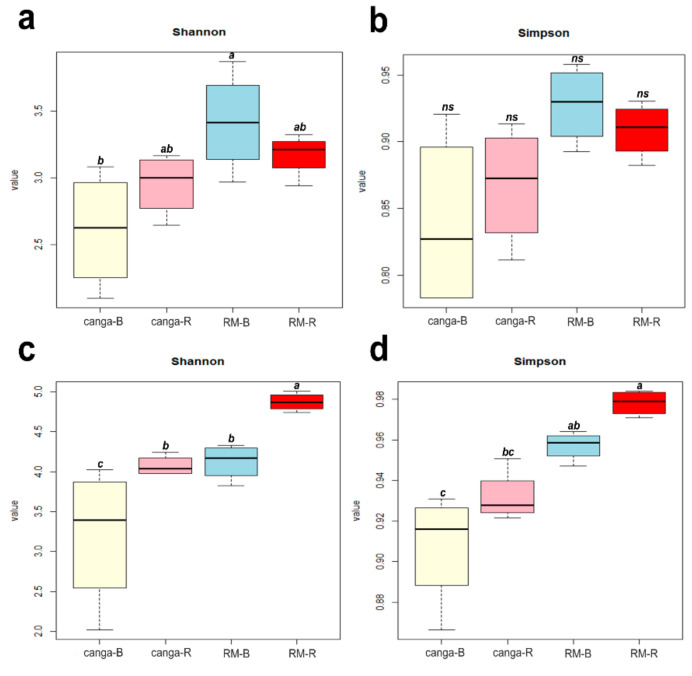
Alpha diversity (Shannon and Simpson) indices of sequences in rhizospheric and bulk substrates associated with *D. apurensis* growing in *canga* or rehabilitating minelands (RM). (**a**,**b**) Indices of fungal 18S rRNA. (**c**,**d**) Indices of bacterial 16S rRNA. canga_B and RM_B, bulk substrate; canga_R and RM_R, rhizospheric substrate. Different lowercase letters indicate statistical differences (*p* > 0.05); ns = not significant).

The ITS2 sequencing produced 3,948,832 raw reads across 16 input libraries. After quality filtering, 2,760,666 amplicon sequences were selected. The number of fungal OTUs in each sample ranged from 359 to 789 and was on average 563 (Table S1). *Canga* soils showed more fungal sequences (1,404,745; comprising 521 ± 132 OTUs) than RM (1,355,921; comprising 605 ± 97 OTUs). Additionally, rhizosphere soil samples presented more sequences (1,531,425; comprising 634 ± 116 OTUs) than the bulk soil samples (1,229,241; comprising 492 ± 78 OTUs). The Shannon and Simpson diversity indexes of fungal sequences were higher in RM, with higher values in the bulk soil substrates of RM samples (Figure 3).

The 16S sequencing produced 4,208,259 raw reads across 16 input libraries. After quality filtering, 1,361,859 sequences were considered. The number of bacterial OTUs ranged from 205 to 996 and was on average 579 (Table S2). The soils from RM showed a higher number of sequences (769,122; comprising 723 ± 240 OTUs) than *canga* (592,737; comprising 435 ± 190 OTUs), whereas more bacterial sequences were detected in the bulk soils (750,740; comprising 601 ± 366) than in the rhizosphere soils (611,119; comprising 556 ± 76 OTUs). The Shannon and Simpson diversity indexes were higher in the RM samples, with higher values in the rhizospheric substrate samples (Figure 3).

In *canga*, the analysis of fungal sequences showed Ascomycota and Basidiomycota as the most abundant phyla in the rhizosphere and bulk soils (Figures S2 and S3, Table S3). Glomeromycota was also detected in bulk soil samples (Figures S2 and S3, Table S3). Regarding bacterial sequences, Acidobacteria, Proteobacteria, and Actinobacteria were the three most abundant phyla in the rhizosphere and bulk soils (Figures S2 and S3, Table S3).

In RM, the analysis of fungal sequences revealed Asc omycota, Basidiomycota, and Glomeromycota as the most dominant phyla in the rhizosphere and bulk soil substrate (Figures S2 and S3, Table S3). Proteobacteria, Acidobacteria, and Actinobacteria were the more abundant phyla in both the rhizosphere and bulk soil substrates (Figures S2 and S3, Table S3).

**Figure 4:**
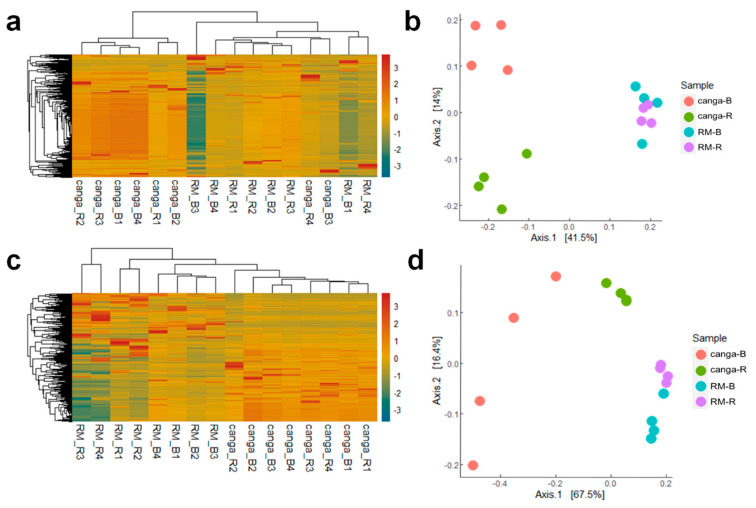
Heatmap and principal coordinate analysis for cumulative sum scaling (CSS)-normalized counts of the fungal 18S rRNA and bacterial 16S rRNA gene sequences obtained from substrates from *D. apurensis* growing in *canga* or rehabilitating minelands (RM). canga_B and RM_B, bulk substrate; *canga*_R and RM_R, rhizospheric substrate. (**a**,**b**) Heatmap showing differences between fungal 18S rRNA (**a**) and principal coordinate analysis for cumulative sum scaling of fungal 18S rRNA (**b**). (**c**,**d**) Heatmap showing differences between bacterial 16S rRNA (**c**) and principal coordinate analysis for cumulative sum scaling of bacterial 16S rRNA (**d**).

A heatmap based on OTU abundance indicates differences between the structure of the microbial communities associated with plants in both ecosystems (RM and native *canga*) (Figure 4a,c). PCoA and cluster analyses of microbial community structure among the different samples showed that microbial communities were distinct between RM and native *canga*. Microbial communities also differed when comparing rhizospheric and bulk soil from *canga* (considering both fungal and bacterial populations) but not in RM (Figure 4b,d).

**Figure 5:**
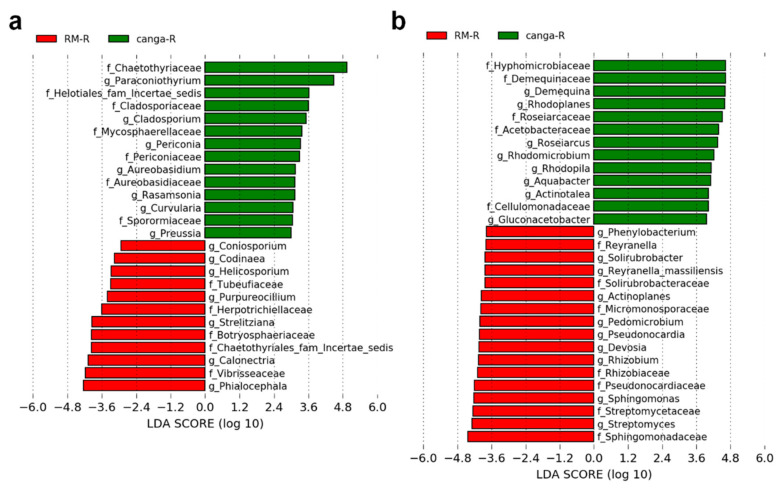
Linear discriminant analysis (LDA) of effect size (LEfSe) to identify preferential taxa in the rhizosphere of plants identified in *canga* (*canga*) or in the rehabilitating minelands (RM). (**a**) Preferential fungal 18S rRNA sequences and (**b**) preferential bacterial 16S rRNA sequences.

Differences in the composition of microbial communities were estimated by calculating LEfSe scores at the family and genus levels. A total of 25 distinct fungal taxa were identified in plants established in *canga* or RM (Figure 5a). Most of these taxa were related to Ascomycota and Basidiomycota. Whereas a total of 29 preferential bacterial taxa were identified in plants growing in *canga* or RM, most of which were related to Proteobacteria and Actinobacteria (Figure 5b).

**Figure 6:**
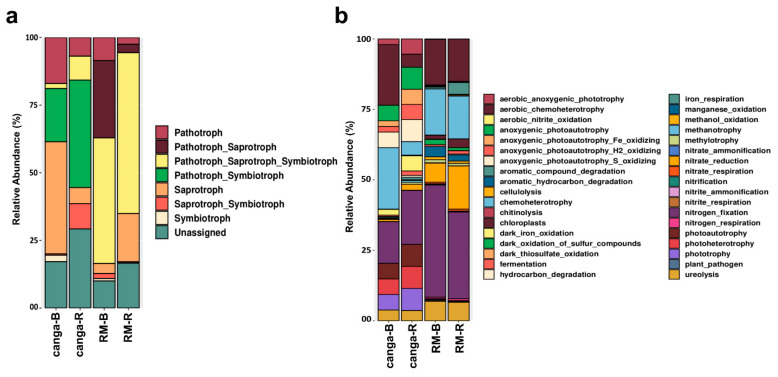
Functional analysis of the communities of 18S rRNA (**a**) and bacterial 16S rRNA (**b**) obtained from *Dioclea apurensis* rhizosphere from plants growing in a *canga* ecosystem (*canga*) or a rehabilitating mineland (RM) in Serra dos Carajás, eastern Amazon.

Seven trophic modes were predicted in the fungal OTUs, being the categories pathotroph_symbiotroph most abundant in the soils from *canga*, whereas the categories pathotroph_saprotroph_symbiotroph were most abundant in the soils from RM (Figure 6). Similarly, 36 predicted functions were identified in the bacterial OTUs, being the function nitrogen fixation and nitrate reduction more abundant in both soils but higher in the RM (Figure 6).

## 3. Discussion

In this study, the proteomic approach has revealed the differential abundances of proteins related to metabolic pathways involved in response to the abiotic stress conditions of both native *canga* and RM. Together, the functional annotation, enrichment, and PPI analyzes showed the co-occurrence of a large part of the essential proteins accumulated in plants growing in each ecosystem and include those related to responses to oxidative stress, drought, P deprivation, excess iron, and symbiosis. Thus, the readiness of a molecular machinery joined to specific rhizosphere-associated fungal and bacterial communities inhabiting the rhizosphere can be considered crucial for establishing this native plant in RM in the eastern Amazon.

### 3.1. Physical and Chemical Properties of Canga and Mining Area Soil Substrates

Alteration in the normal levels of reactive oxygen species (ROS) is one of the main metabolic responses observed in plants growing under stressful environmental conditions [19]. In order to mediate the balance of ROS, the removal system relies on the action of enzymatic and non-enzymatic antioxidants [20]. In this study, proteins involved in synthesizing enzymatic antioxidants such as superoxide dismutase, peroxidases, catalases, glutathione reductase, and monodehydroascorbate reductase showed more abundance in the roots from *canga* (Dataset S1). Additionally, the non-enzymatic antioxidant systems proline-rich receptor protein kinase and betaine aldehyde dehydrogenase (BADH) were more accumulated in the roots from *canga* (Dataset S1). Such results reveal the abundances of proteins involved in the antioxidant system, especially in the roots growing under the severe conditions of *canga*, which also are identified in plants growing in RM.

### 3.2. Proteins Involved in the Response to Water Deficit

Two types of aquaporins showed more abundance in plants from *canga*, including aquaporin PIP1-1 and water stress-induced tonoplast intrinsic protein (Dataset S1), which play a regulatory role in the cellular transport of water in plasma membranes and tonoplasts, respectively [21]. These proteins have been described as essential for improving plant growth, water deficit tolerance, and osmotic balance [22,23]. Roots from *canga* showed higher levels of proteins involved in abscisic acid (ABA) transport and signaling (i.e., phosphatase 2C, ABC transporter, ATP-binding protein, ABC transporter B, and ABC transporter I), as well as proteins involved in signaling by serine/threonine kinase (i.e., phospholipase D alpha 1, proline-rich receptor-like protein kinase and receptor-like protein kinase S) than roots from RM (Dataset S1). ABA has been classified as a versatile phytohormone involved in plant signaling in response to drought stress [24]. Under conditions of water deficit (a common characteristic of *canga* ecosystems), there is also a high levels of phosphatase 2C (PPC2)-type proteins, which acts on the dephosphorylation of SnRK2, maintaining optimal ABA levels during water deficit [25], which agree with the results obtained in the roots from plants growing in *canga*.

High levels of osmolytes such as glycine betaine (GB), which is synthesized in a pathway in which BADH acts as a critical enzyme, has been recently described in plants growing under abiotic stress, including the water deficit [26], contributing to a higher relative water content, the integrity of cell membranes, stabilization of proteins, and detoxification [27]. The relationship of high levels of BADH biosynthesis with the increase in GB levels and greater tolerance to water deficit has already been observed in plants such as *Arabidopsis thaliana* [27], *Sesuvium portulacastrum* [28], and *Zea mays* [29]. In addition to BADH, roots from *canga* showed higher levels of proline-rich receptor-like protein kinase and spermidine synthase (Dataset S1). These proteins have been considered essential components of the cell membrane, playing essential roles in signal transduction in response to drought and stomata activity to prevent water loss [30,31]. The greater abundance of these water deficit-responsive proteins in roots from *canga* indicates that *D. apurensis* have developed adaptive mechanisms to resist extensive drought periods in *canga* and help resist the drought events in RM.

### 3.3. Proteins Involved in the Response to Metal Stress

Metal stress directly influences the establishment and growth of plants in mining areas. In fact, soils from *canga* showed higher levels of Fe (Table 1). High levels of metals in the soil solution can activate distinct signaling pathways such as cadmium-dependent, mitogen-activated protein kinase, ROS, and phytohormones, enhancing the expression of transcription factors or stress-responsive genes [32]. Most of these proteins were more accumulated in roots from *canga* (Dataset S1). Protein kinases, calmodulins, and calcium-dependent protein kinases have been described as receptors capable of activating signaling networks in the tolerance to the excess of metals [33,34].

Additionally, Glutathione-related proteins are directly involved in the balance of metals in the intracellular medium, a process that has been described in *Cucumis sativus*, *Triticum aestivum*, and *Beta vulgaris* [35,36]. These results also agree with Jiang et al. [37], who reported a direct role of heat shock proteins (HSP70) in the contribution of tolerance to metal stress in *Rosa hybrida*. Such proteins were more accumulated in roots from *canga* (Dataset S1). Similarly, proteins related to the biosynthesis of compounds commonly detected in plants growing under metal stress, such as phenylalanine ammonia-lyase and nicotianamine synthase, were detected in this study (Dataset S1) [38,39,40]. Additionally, ferritin was also more accumulated in roots from *canga*, and their role in cytoplasmatic sequestration of soluble iron [38,39] can contribute to the diminishing of the negative effects of the metal in *D. apurensis*.

### 3.4. Proteins Involved in the Response to P-Starvation

According to this study, P levels in RM were higher than in *canga* (Table 1). The higher concentration of P in RM can be explained by hydroseeding containing NPK fertilization in these sites. Phosphorus deficiency in *canga* can be related to the formation of complexes between P and iron oxides, limiting plant uptake availability [41]. Roots from plants sampled in *canga* showed more proteins related to P depletion, such as monogalactosyldiacylglycerol synthase 2, phospholipase, alcohol dehydrogenase, extracellular purple acid phosphatases, and antioxidant system proteins such as catalases and monodehydroascorbate reductases [42,43,44]. During Pi deprivation, glycolipids are transferred to extraplastid membranes, where they replace degraded phospholipids to meet the need for Pi essential for various biological processes in the cell. This membrane remodeling occurs in a process dependent on the activation of monogalactosyldiacylglycerol synthase induced by a low Pi level. Studies have already observed this pattern in leaves and roots with different plant species under Pi depletion, including *Sesamum indicum*, *Zea mays*, and *Arabidopsis thaliana* [42,45,46].

Additionally, three phospholipase D alpha-1 proteins were also more accumulated in roots from *canga* (Dataset S1). Phospholipase induction has been observed in studies with plants during P starvation [43]. These proteins are involved in P storage and induction of root growth to tolerate P scarcity conditions [43], such as in *canga*. In this sense, *D. apurensis* from *canga* synthesize proteins that support the growth under low P levels or induce mineralization from the soil substrate in *canga*. This study showed that alcohol dehydrogenase (ADH) was more accumulated in roots from *canga* (Dataset S1). The increase in ADH levels is in line with the results observed in studies with different plant species growing under P shortage. The high levels of this protein is observed together with increases in glycolysis and fermentation intermediaries, possibly related to cell expansion in Pi-limitation [47,48]. In acidic and Fe-rich soil substrates, such as the *canga* ecosystems, P can form complexes with iron oxides in an inaccessible form for absorption by plant roots [44]. This study identified extracellular purple acid phosphatases in roots from *canga* (Dataset S1). These proteins belong to a group of hydrolases induced by P deficiency, acting in recycling P from esters and anhydrides [44]. Additionally, P scarcity in *canga* soil substrates can induce ROS formation, which was also observed in the roots from *canga* (Dataset S1) participating in the signaling of cellular responses and protein biosynthesis involved in adaptations to P starvation [49]. Under both conditions, the roots expressed transcription factors commonly reported under P shortage, including MYB, WRKY, and HLH (Dataset S1), which regulate the expression of target genes and define metabolic responses to P starvation [50,51]. The set of proteins accumulated in plants growing in *canga* should contribute to the adaptation of *D. apurensis* in this ecosystem, although there are still no impediments to its development in environments with optimal P levels as in RM.

### 3.5. Proteins Involved in Symbiosis

Under abiotic stress, plants depend on beneficial soil microorganisms to become established in harmful environments such as *canga*. In this study, the analyses have detected several proteins involved in the establishment of symbiosis with bacteria and fungi, including nodulins [52], monodehydroascorbate ascorbate reductase [53], and enzymes involved in the ascorbate-glutathione cycle [54]. These enzymes were detected in the roots from both ecosystems, with higher levels in the roots from *canga* (Dataset S1). The nodulation in *D. apurensis* has been observed in recent studies with depletion of nutrients such as N [7]. Considering the successful establishment of *D. apurensis* in *canga*, these species contribute to maintaining essential soil processes such as N fixation during mineland rehabilitation [7,55]. The higher levels of symbiosis-related proteins in the roots from *canga* than from RM is explained by the higher diversity of plant species found in *canga* ecosystems and their corresponding effect on soil microbial communities.

### 3.6. Rhizosphere-Associated Microbial Communities

The rhizosphere-associated fungal and bacterial community analysis showed that specific taxa are enriched in the rhizosphere of *D. apurensis*. Proteobacteria and Ascomycota were the most abundant bacterial and fungal phyla, respectively, in both rhizosphere and bulk soil substrates (Figure 5). Recent studies have reported similar results in plants growing in ecosystems affected by mining activities, where the soil microbial communities and specific mechanisms of abiotic stress tolerance can be considered key to promoting the phytostabilization towards rehabilitation of ecosystems services [8,56]. Among the preferential microorganisms identified in this study, several beneficial saprophytic, free-living, symbiotic, and endophytic taxa were directly identified in the rhizospheric soil, especially in plants from *canga*, where specific fungal and bacterial taxa belonging to plant growth-promoting microorganisms were detected (e.g., *Paraconiothyrium*, *Rasamsonia*, *Scytalidium*, *Rhodoplanes*, *Bradyrhizobium*, *Rhizobium*, *Roseiarcus*, and *Actinotalea*) (Table S3; Figure 5). Such results agree with recent studies analyzing the diversity of microbial communities associated with plants growing under stressful environmental conditions, where specific rhizosphere-associated microorganisms have been described as essential to promote plant establishment [57,58]. This study has also detected a higher microbial diversity in plants from RM (especially fungal taxa), which can be related to the presence of widespread soil microorganisms inhabiting the RM soil substrates, with competition-mediated co-existence, latent soil microbes, and low influence of plant species on the soil microbiota (compared to native *canga*). However, the predicted functions of microorganisms involved in N-fixation were more abundant in RM than *canga* (Figure 6), showing that low specificity for microbial taxa can also be a characteristic supporting the establishment of this species in RM.

The rhizosphere-associated fungal and bacterial communities play an essential role in establishing native species in RM [59,60]. Additionally, several beneficial bacterial and fungal genera with key roles in nutrient solubilization, plant growth promotion, and defense against phytopathogens were detected (e.g., Glomeromycetes, *Sphingomonas*, *Actinotalea*, *Rhizobium*, *Rasamsonia*, *Paraconiothyrium*) [61,62,63]. Despite the higher diversity of rhizosphere-associated microorganisms identified in plants growing in RM, they were mostly different from those detected in *canga*, denoting that *D. apurensis* can associate with beneficial soil microorganisms inhabiting soil substrates in RM without an apparent specificity, similar to what has been described in *Mimosa acutistipula* [8]. Accordingly, the specific rhizosphere-associated microbial communities of *D. apurensis*, which is related to better performance under abiotic stress conditions in *canga* [64], must be considered in mineland rehabilitation projects to preserve essential microbe-mediated processes that can contribute to later successional stages of rehabilitation in Amazonian *canga*.

## 4. Materials and Methods

### 4.1. Soil Substrate Sampling and Chemical Analyses

Samples were collected at the end of the wet season (May 2018) in a native metalliferous savanna in Serra dos Carajás, Pará, northern Brazil. The sampling sites included: (i) a native herbaceous shrub *canga* ecosystem with minimal anthropogenic intervention (6°00′41.0″ S 50°17′45.0″ W); and (ii) a RM in waste piles of iron mining (6°02′32.0″ S 50°07′04.0″ W), where a revegetation program started in 2014. The application of *D. apurensis* seeds was carried out by hydroseeding containing NPK fertilization with 04-14-08 (8 kg for each 8 × 12 m of soil substrate).

The region’s climate is tropical warm with a rainy season from November to March and a dry season from May to September, an average annual rainfall of 2033 mm, and temperatures varying between 25.1 °C and 26.3 °C [9,65]. Bulk soil samples (n = 5) were collected near *D. apurensis* roots naturally growing in *canga* and from *D. apurensis* growing in an RM project, at a depth of 10 cm, 5 m away from each plant considering zones without other plants. Rhizospheric soil (n = 5) was taken by gently shaking the soil substrate adhered to the root system from plants growing in *canga* and RM. The samples of bulk soil were submitted to chemical and physical analyses. After being air-dried, the samples were sieved using a 2 mm mesh. The pH was determined in a 1:2.5 soil substrate to water ratio, and the organic carbon content was determined using the potassium dichromate (K_2_Cr_2_O_7_) method. The available P, K, B, Zn, Fe, Mn, and Cu were determined using the Mehlich-1 method (0.05 mol L^−1^ HCl + 0.0125 mol L^−1^ H_2_SO_4_), S-SO_4_^−2^ by calcium phosphate monobasic at 0.01 M, and the total N content was determined using the Kjeldahl method [66]. The soil texture was determined as described by Kettler et al. [67].

### 4.2. Root Sampling and Protein Isolation

Roots from five *D. apurensis* individual plants were collected at each sampling site, kept in a cold phenol/SDS (sodium dodecyl sulfate) buffer, and transported to the laboratory for further processing. Protein isolation, determination of protein concentrations, and further processing were performed according to the protocol proposed by do Nascimento et al. [68], with minor modifications (Table S4).

### 4.3. Proteome Analysis

The identification and quantification of proteins were performed in a nanoACQUITY UPLC^®^ ultra-performance liquid chromatography (Milford, MA, USA), configured for fractionation in two dimensions as reported in Herrera et al. [69]. Five micrograms of the peptides were analyzed with five analytical replicates. The first dimension used a 5 μm XBridge BEH130 C18 (300 μm × 50 mm) and a Symmetry C18 5 μm (180 μm × 20 mm) trapping column at a flow rate of 2000 μL min^−1^. The second dimension used a 1.7 μm BEH130 C18 1.8 μm (100 μm × 100 mm) analytical column, at a flow rate of 400 μL min^−1^. The samples were separated in five fractions with a gradient of 10.8, 14.0 16.7, 20.4, and 65.0% acetonitrile. The chromatograph was coupled to a NanoLock ESI-Q-ToF SYNAPT G2-S (Waters) mass spectrometer. The acquisition ranged from 50 to 2000 Da, in MS^E^ mode (data independent analysis) at a scan rate of 0.5 s and an interscan delay of 0.1 s.

The data were processed using the Progenesis QI software (Waters) for identification and quantification, using the Viridiplantae database from UniProt (UniProtKB/swiss-prot, uniprot.org, accessed on 10 October 2021). Protein identification was accepted if the probability of identifying peptides was greater than 90% and proteins with 95%. The significance levels of the differential abundances of proteins were determined by applying the ANOVA test (*p* < 0.05). To compare the proteome of *D. apurensis* grown in *canga* or RM, a PCA of the proteins with differential abundances and with *p* < 0.05 were produced in the R software v3.6.3 (R Core Team 2018; https://www.R-project.org, accessed on 10 October 2021), using packages FactoMineR, Factoshiny, and Factoextra. The functional annotation of proteins was performed using the OmicsBox v1.2.4 (bioBam) and Uniprot (UniProtKB/swiss-prot, uniprot.org, accessed on 10 October 2021). Kyoto Encyclopedia of Gene and Genome (KEGG) pathway enrichment analysis for proteins was performed using the KOBAS software v3.0. [70] (kobas.cbi.pku.edu.cn, accessed on 10 October 2021) with *Arabidopsis thaliana* as the background species for the analysis. The R package ggplot2 was used for the enriched KEGG pathways visualization. The PPI networks were predicted based on functional analysis using the software STRING (research tool for recovering genes/proteins in interaction) v11.0 (http://string-db.org/, accessed on 10 October 2021), using homologous proteins from the *Arabidopsis thaliana* as the background species.

### 4.4. Microbial Diversity

Total DNA was extracted from 0.25 g of soil using the PowerSoil DNA Isolation Kit (QIAGEN, Hilden, Germany), according to the manufacturer’s recommendations. The DNA concentration was determined with the Qubit fluorometer (Thermo Fisher Scientific, Waltham, MA, USA), and the quality was verified in a 1 % electrophoresis agarose gel.

The amplicon libraries for bacteria and fungi were prepared according to Costa et al. [8], with minor modifications. The 16S rRNA gene was amplified by PCR using the bacterial primer set S-D-Bact-0341-b-S-17-N (5′-TCGTCGGCAGCGTCAGATGTGTATAAGAGACAGCCTACGGGNGGCWGCAG-3′) and S-D-Bact-0785-a-A-21-N (5′-GTCTCGTGGGCTCGGAGATGTGTATAAGAGACAGGACTACHVGGGTATCTAATCC-3′). After a hot start at 95 °C for 3 min, 35 PCR amplification cycles at 95 °C for 30 seg, 55 °C for 30 seg, and 72 °C for 30 seg were performed, followed by a final extension step at 72 °C for 5 min. The ITS region of the 18S rRNA gene was amplified by PCR using the primer set fITS7i (5′-TCGTCGGCAGCGTCAGATGTGTATAAGAGACAGGTGARTCATCGAATCTTTG-3′) and ITS4i (5′-GTCTCGTGGGCTCGGAGATGTGTATAAGAGACAGTCCTCCGCTTATTGATATGC-3′). After a hot start at 94 °C for 2 min, 35 PCR amplification cycles at 94 °C for 30 seg, 56 °C for 1 min, and 72 °C for 30 seg were performed, followed by a final extension step at 72 °C for 7 min.

The size and quality of the PCR fragments were estimated on an Agilent 2100 Bioanalyzer (Agilent Technologies, Santa Clara, CA, USA) using a DNA 1000 chip. The libraries were purified with the AMPure XP purification kit (Beckman Coulter, Brea, CA, USA) and further processed with the Nextera XT kit (Illumina, San Diego, CA, USA). The gene libraries were sequenced in a Miseq-Illumina platform using a MiSeq V3 reagent kit (600 cycles; Illumina) in the human and medical genetics laboratory at Universidade Federal do Pará (Belém, PA, Brazil).

The ITS and 16S sequences were analyzed using the Pipeline for MetaBarcoding Analysis (PIMBA), which allows the analysis of metabarcodes based on the pipeline QIIME [71]. The low-quality sequences were filtered and trimmed using PRINSEQ v0.20.4, and forward and reverse sequences were merged using PEAR v0.9.19 [72]. Reads were dereplicated, singletons removed, and the sequences were truncated to 200 for fungi and 240 for bacteria. Chimeras were filtered, and the sequences were grouped into operative taxonomic units (OTUs) using VSEARCH v2.8.2. The taxonomic assignment was developed using the UNITE database for fungi and the Ribosomal Database Project for bacteria [73,74]. Graphs were constructed considering the alpha and beta diversity in R software using the ggplot2 and vegan packages. Beta diversity was calculated, and principal coordinate analysis (PCoA) graphs were constructed using the “weighted UniFrac distances” in R software using the phyloseq package. Heatmaps were constructed with the total abundance of OTUs using R software (packages pheatmap and phyloseq). Alpha diversity was estimated using the vegan package with the Shannon and Simpson diversity indices. Additionally, clustering analyses of the data were performed using an “hclust” function with the options “methods = ward.D2” and “method.dist = correlation” from the pvclust package in the R software. Permutational multivariate analysis of variance was applied using the function “adonis” (vegan package). Linear discriminant analysis (LDA) of effect size (LEfSe) was performed with the Kruskal–Wallis test, and the effect size was estimated with a logarithmic score of 2.0 in the LDA. Finally, the predicted ecological roles of the identified microbial taxa were assigned using FUNGuild v1.1 [75] and FAPROTAX v1.2.4 [76] in Python v3.8.2. The data were plotted in the R software using the viridis, dplyr, and scales packages.

## 5. Conclusions

This study showed that *D. apurensis* growing in native *canga* have a set of proteins involved in the response to environmental stress to cope with the abiotic stress challenges. Its ability to increase the levels of a wide range of proteins in response to the challenging conditions of post-mining areas enhances *D. apurensis* establishment in rehabilitating minelands. Among them, the identification of proteins involved in the antioxidant system, response to water deficit, excess of metals, and deficiency of P. Additionally, our results confirm that *D. apurensis* establish interactions with beneficial microbial taxa without specificity. High levels of specific proteins involved in response to severe environmental conditions and interaction with key microbes at the rhizosphere are characteristics that can be identified in native species to select and diversify the plant species used for mineland rehabilitation.

## Figures and Tables

**Table 1:** Physical and chemical characteristics of soils associated with *Dioclea apurensis* growing in *canga* and rehabilitating minelands (RM). Soil results are mean ± standard deviation for n = 5.

	RM	*Canga*
Clay (g kg^−1)^	486.7 ± 37.2	287.5 ± 103
Silt (g kg^−1)^	158.3 ± 40.8	87.5 ± 75
Sand (g kg^−1)^	355 ± 68.9	625 ± 177
pH H_2_O	6.1 ± 0.3	4.7 ± 0.6
pH CaCl_2_	5.3 ± 0.2	4.2 ± 0.3
Available P (mg dm^−3^)	19.5 ± 15.9	1 ± 0.9
Total N (dag kg^−1^)	0.1 ± 0.06	0.4 ± 0.3
Organic matter (dag kg^−1^)	1.4 ± 0.6	7.7 ± 0.9
Na (mg dm^−3^)	4.7 ± 0.6	11.4 ± 6.2
K (mg dm^−3^)	22.6 ± 6.8	22.7 ± 6.8
B (mg dm^−3^)	0.1 ± 0.03	0.2 ± 0.08
Cu (mg dm^−3^)	0.6 ± 0.3	1.8 ± 0.5
Fe (mg dm^−3^)	11.3 ± 6.3	372 ± 92
Mn (mg dm^−3^)	59.7 ± 9.4	2.8 ± 1.5
Zn (mg dm^−3^)	2.1 ± 1.6	1.4 ± 0.9

## Data Availability

The sequences obtained in this study were deposited in the NCBI Sequence Read Archive (https://www.ncbi.nlm.nih.gov/sra/PRJNA690164, accessed on 1 June 2021) under the accession number PRJNA690164. The proteomic data was submitted to Massive repository under the accession MSV000087423 (https://massive.ucsd.edu/, accessed on 1 June 2021).

## Supplementary Materials

The following are available online at https://www.mdpi.com/article/10.3390/plants11050712/s1. Figure S1: Top 40 categories obtained by functional annotation in different biological processes. (a) Most abundant proteins in *canga* plants and (b) the most abundant proteins in rehabilitating mineland (RM) plants; Figure S2: Relative abundance at the phylum level of major fungal (A) and bacterial (B) sequences associated with the rhizosphere (R) or bulk soil (B) of *Dioclea apurensis* growing in *canga* (*canga*) or a rehabilitating mineland (RM) in Serra dos Carajás, eastern Amazon (n = 4); Figure S3: Relative abundance at the genus level of major fungal (A) and bacterial (B) sequences associated with the rhizosphere (R) or bulk soil (B) of *Dioclea apurensis* growing in *canga* (*canga*) or a rehabilitating mineland (RM) in Serra dos Carajás, eastern Amazon (n = 4); Table S1: Fungal 18S rRNA sequences obtained in rhizospheric and bulk substrates samples from *Dioclea apurensis* growing in *canga* (*canga*) or rehabilitating minelands (RM); Table S2: Bacterial 16S rRNA sequences obtained in rhizospheric and bulk substrates samples from *Dioclea apurensis* growing in *canga* (*canga*) or rehabilitating minelands (RM); Table S3: Most abundant fungal and bacterial taxa identified in *Dioclea apurensis* soil substrates in plants from *canga* (*canga*) and rehabilitating minelands (RM). Numbers between parentheses are the relative abundance percentage (RA %) of each identified taxa; Table S4: Steps and procedures of protein extraction; Dataset S1: Protein report of 396 proteins with differential abundances in *canga* and RM plants filtered based on *p* < 0.05 and fold change ≥ 1.5; Dataset S2: PPI enrichment of *Dioclea apurensis* root proteins in STRING.

## References

[1] dos Santos R.S.P., Milanez B. (2015). The Global Production Network for iron ore: Materiality, corporate strategies, and social contestation in Brazil. Extr. Ind. Soc..

[2] Jacobi C.M., Do Carmo F.F., Vincent R.C., Stehmann J.R. (2007). Plant communities on ironstone outcrops: A diverse and endangered Brazilian ecosystem. Biodivers. Conserv..

[3] Skirycz A., Castilho A., Chaparro C., Carvalho N., Tzotzos G., Siqueira J.O. (2014). *Canga* biodiversity, a matter of mining. Front. Plant Sci..

[4] Lehmann J., da Silva Cravo M., de Macêdo J.L.V., Moreira A., Schroth G. (2001). Phosphorus management for perennial crops in central Amazonian upland soils. Plant Soil.

[5] Gastauer M., Souza Filho P.W.M., Ramos S.J., Caldeira C.F., Silva J.R., Siqueira J.O., Neto A.E.F. (2019). Mine land rehabilitation in Brazil: Goals and techniques in the context of legal requirements. Ambio.

[6] Carvalho C.S., Forester B.R., Mitre S.K., Alves R., Imperatriz-Fonseca V.L., Ramos S.J., Resende-Moreira L.C., Siqueira J.O., Trevelin L.C., Caldeira C.F. (2021). Combining genotype, phenotype, and environmental data to delineate site-adjusted provenance strategies for ecological restoration. Mol. Ecol. Resour..

[7] Ramos S.J., Gastauer M., Mitre S.K., Caldeira C.F., Silva J.R., Neto A.E.F., Oliveira G., Souza Filho P.W., Siqueira J.O. (2020). Plant growth and nutrient use efficiency of two native Fabaceae species for mineland revegetation in the eastern Amazon. J. For. Res..

[8] Costa P.H.d.O., Nascimento S.V.d., Herrera H., Gastauer M., Ramos S.J., Caldeira C.F., Oliveira G., Valadares R.B.d.S. (2021). Non-Specific Interactions of Rhizospheric Microbial Communities Support the Establishment of *Mimosa acutistipula* var. *ferrea* in an Amazon Rehabilitating Mineland. Processes.

[9] Gastauer M., de Medeiros Sarmento P.S., Santos V.C.A., Caldeira C.F., Ramos S.J., Teodoro G.S., Siqueira J.O. (2020). Vegetative functional traits guide plant species selection for initial mineland rehabilitation. Ecol. Eng..

[10] Queiroz L.P.d. (2009). Leguminosas da Caatinga.

[11] Ramos S.J., Caldeira C.F., Gastauer M., Costa D.L.P., Neto A.E.F., de Souza F.B.M., Souza-Filho P.W.M., Siqueira J.O. (2019). Native leguminous plants for mineland revegetation in the eastern Amazon: Seed characteristics and germination. New For..

[12] Giannini T.C., Giulietti A.M., Harley R.M., Viana P.L., Jaffe R., Alves R., Pinto C.E., Mota N.F., Caldeira C.F., Imperatriz-Fonseca V.L. (2017). Selecting plant species for practical restoration of degraded lands using a multiple-trait approach. Austral Ecol..

[13] Aggangan N.S., Anarna J.A., Cadiz N.M. (2019). Tree legume–microbial symbiosis and other soil amendments as rehabilitation strategies in mine tailings in the Philippines. Philipp. J. Sci..

[14] Jasper D.A. (2007). Beneficial soil microorganisms of the jarrah forest and their recovery in bauxite mine restoration in Southwestern Australia. Restor. Ecol..

[15] de Oliveira-Longatti S.M., Marra L.M., Lima Soares B., Bomfeti C.A., Da Silva K., Avelar Ferreira P.A., de Souza Moreira F.M. (2014). Bacteria isolated from soils of the western Amazon and from rehabilitated bauxite-mining areas have potential as plant growth promoters. World J. Microbiol. Biotechnol..

[16] Oliveira Silva A., Azarias Guimarães A., da Costa A.M., Louzada Rodrigues T., de Soares Carvalho T., Reis Sales F., de Souza Moreira F.M. (2020). Plant growth-promoting rhizobacterial communities from an area under the influence of iron mining and from the adjacent phytophysiognomies which have high genetic diversity. Land Degrad. Dev..

[17] Herrera H., Fuentes A., Ortiz J., Soto J., da Silva Valadares R.B., Salas-Eljatib C., Arriagada C. (2022). Root-associated endophytes isolated from juvenile *Ulex europaeus* L. (Fabaceae) plants colonizing rural areas in South-Central Chile. Plant Soil.

[18] Lugtenberg B.J., Malfanova N., Kamilova F., Berg G. (2013). Plant growth promotion by microbes. Mol. Microb. Ecol. Rhizosphere.

[19] Xie H., Yang D.-H., Yao H., Bai G., Zhang Y.-H., Xiao B.-G. (2016). iTRAQ-based quantitative proteomic analysis reveals proteomic changes in leaves of cultivated tobacco (*Nicotiana tabacum*) in response to drought stress. Biochem. Biophys. Res. Commun..

[20] Ahmad P., Jaleel C.A., Salem M.A., Nabi G., Sharma S. (2010). Roles of enzymatic and nonenzymatic antioxidants in plants during abiotic stress. Crit. Rev. Biotechnol..

[21] Li R., Wang J., Li S., Zhang L., Qi C., Weeda S., Zhao B., Ren S., Guo Y.-D. (2016). Plasma membrane intrinsic proteins SlPIP2; 1, SlPIP2; 7 and SlPIP2; 5 conferring enhanced drought stress tolerance in tomato. Sci. Rep..

[22] Caldeira C.F., Jeanguenin L., Chaumont F., Tardieu F. (2014). Circadian rhythms of hydraulic conductance and growth are enhanced by drought and improve plant performance. Nat. Commun..

[23] Paudel I., Gerbi H., Zisovich A., Sapir G., Ben-Dor S., Brumfeld V., Klein T. (2019). Drought tolerance mechanisms and aquaporin expression of wild vs. cultivated pear tree species in the field. Environ. Exp. Bot..

[24] González-Villagra J., Kurepin L.V., Reyes-Díaz M.M. (2017). Evaluating the involvement and interaction of abscisic acid and miRNA156 in the induction of anthocyanin biosynthesis in drought-stressed plants. Planta.

[25] Baek D., Kim M.C., Kumar D., Park B., Cheong M.S., Choi W., Park H.C., Chun H.J., Park H.J., Lee S.Y. (2019). AtPR5K2, a PR5-like receptor kinase, modulates plant responses to drought stress by phosphorylating protein phosphatase 2Cs. Front. Plant Sci..

[26] Wang F., Wang M., Guo C., Wang N., Li X., Chen H., Dong Y., Chen X., Wang Z., Li H. (2016). Cloning and characterization of a novel betaine aldehyde dehydrogenase gene from *Suaeda corniculata*. Genet. Mol. Res..

[27] Golestan Hashemi F.S., Ismail M.R., Rafii M.Y., Aslani F., Miah G., Muharam F.M. (2018). Critical multifunctional role of the betaine aldehyde dehydrogenase gene in plants. Biotechnol. Biotechnol. Equip..

[28] Yang C., Zhou Y., Fan J., Fu Y., Shen L., Yao Y., Li R., Fu S., Duan R., Hu X. (2015). SpBADH of the halophyte Sesuvium portulacastrum strongly confers drought tolerance through ROS scavenging in transgenic Arabidopsis. Plant Physiol. Biochem..

[29] Zhang L., Gao M., Hu J., Zhang X., Wang K., Ashraf M. (2012). Modulation role of abscisic acid (ABA) on growth, water relations and glycinebetaine metabolism in two maize (*Zea mays* L.) cultivars under drought stress. Int. J. Mol. Sci..

[30] Satish L., Rency A.S., Ramesh M. (2018). Spermidine sprays alleviate the water deficit-induced oxidative stress in finger millet (*Eleusine coracana* L. Gaertn.) plants. 3 Biotech.

[31] Bai L., Zhang G., Zhou Y., Zhang Z., Wang W., Du Y., Wu Z., Song C.P. (2009). Plasma membrane-associated proline-rich extensin-like receptor kinase 4, a novel regulator of Ca^2+^ signalling, is required for abscisic acid responses in *Arabidopsis thaliana*. Plant J..

[32] Tiwari S., Lata C. (2018). Heavy metal stress, signaling, and tolerance due to plant-associated microbes: An overview. Front. Plant Sci..

[33] Jalmi S.K., Bhagat P.K., Verma D., Noryang S., Tayyeba S., Singh K., Sharma D., Sinha A.K. (2018). Traversing the links between heavy metal stress and plant signaling. Front. Plant Sci..

[34] Zhao D., Li T., Wang J., Zhao Z. (2015). Diverse strategies conferring extreme cadmium (Cd) tolerance in the dark septate endophyte (DSE), *Exophiala pisciphila*: Evidence from RNA-seq data. Microbiol. Res..

[35] Erbasol I., Bozdag G.O., Koc A., Pedas P., Karakaya H.C. (2013). Characterization of two genes encoding metal tolerance proteins from *Beta vulgaris* subspecies maritima that confers manganese tolerance in yeast. Biometals.

[36] Vatansever R., Filiz E., Eroglu S. (2017). Genome-wide exploration of metal tolerance protein (MTP) genes in common wheat (*Triticum aestivum*): Insights into metal homeostasis and biofortification. Biometals.

[37] Jiang C., Bi Y., Zhang R., Feng S. (2020). Expression of RcHSP70, heat shock protein 70 gene from Chinese rose, enhances host resistance to abiotic stresses. Sci. Rep..

[38] Kottmann L., Wilde P., Schittenhelm S. (2016). How do timing, duration, and intensity of drought stress affect the agronomic performance of winter rye?. Eur. J. Agron..

[39] Shevyakova N., Eshinimaeva B.T., Kuznetsov V.V. (2011). Expression of ferritin gene in *Mesembryanthemum crystallinum* plants under different supply with iron and different intensity of oxidative stress. Russ. J. Plant Physiol..

[40] Stein R.J., Ricachenevsky F.K., Fett J.P. (2009). Differential regulation of the two rice ferritin genes (OsFER1 and OsFER2). Plant Sci..

[41] Fink J.R., Inda A.V., Tiecher T., Barrón V. (2016). Iron oxides and organic matter on soil phosphorus availability. Cienc. Agrotecnol..

[42] Wang F., Ding D., Li J., He L., Xu X., Zhao Y., Yan B., Li Z., Xu J. (2020). Characterisation of genes involved in galactolipids and sulfolipids metabolism in maize and Arabidopsis and their differential responses to phosphate deficiency. Funct. Plant Biol..

[43] Lin D.L., Yao H.Y., Jia L.H., Tan J.F., Xu Z.H., Zheng W.M., Xue H.W. (2020). Phospholipase D-derived phosphatidic acid promotes root hair development under phosphorus deficiency by suppressing vacuolar degradation of PIN-FORMED2. New Phytol..

[44] Tran H.T., Hurley B.A., Plaxton W.C. (2010). Feeding hungry plants: The role of purple acid phosphatases in phosphate nutrition. Plant Sci..

[45] Shimojima M., Watanabe T., Madoka Y., Koizumi R., Yamamoto M.P., Masuda K., Yamada K., Masuda S., Ohta H. (2013). Differential regulation of two types of monogalactosyldiacylglycerol synthase in membrane lipid remodeling under phosphate-limited conditions in sesame plants. Front. Plant Sci..

[46] Shimojima M., Madoka Y., Fujiwara R., Murakawa M., Yoshitake Y., Ikeda K., Koizumi R., Endo K., Ozaki K., Ohta H. (2015). An engineered lipid remodeling system using a galactolipid synthase promoter during phosphate starvation enhances oil accumulation in plants. Front. Plant Sci..

[47] Alexova R., Millar A.H. (2013). Proteomics of phosphate use and deprivation in plants. Proteomics.

[48] Chevalier F., Rossignol M. (2011). Proteomic analysis of *Arabidopsis thaliana* ecotypes with contrasted root architecture in response to phosphate deficiency. J. Plant Physiol..

[49] Muneer S., Jeong B.R. (2015). Proteomic analysis provides new insights in phosphorus homeostasis subjected to pi (inorganic phosphate) starvation in tomato plants (*Solanum lycopersicum* L.). PLoS ONE.

[50] Zhou J., Jiao F., Wu Z., Li Y., Wang X., He X., Zhong W., Wu P. (2008). OsPHR2 is involved in phosphate-starvation signaling and excessive phosphate accumulation in shoots of plants. Plant Physiol..

[51] Aleksza D., Horváth G.V., Sándor G., Szabados L. (2017). Proline accumulation is regulated by transcription factors associated with phosphate starvation. Plant Physiol..

[52] Sakamoto K., Ogiwara N., Kaji T., Sugimoto Y., Ueno M., Sonoda M., Matsui A., Ishida J., Tanaka M., Totoki Y. (2019). Transcriptome analysis of soybean (*Glycine max*) root genes differentially expressed in rhizobial, arbuscular mycorrhizal, and dual symbiosis. J. Plant Res..

[53] Vadassery J., Tripathi S., Prasad R., Varma A., Oelmüller R. (2009). Monodehydroascorbate reductase 2 and dehydroascorbate reductase 5 are crucial for a mutualistic interaction between Piriformospora indica and Arabidopsis. J. Plant Physiol..

[54] Luo S., Yin J., Peng Y., Xie J., Wu H., He D., Li X., Cheng G. (2020). Glutathione is Involved in Detoxification of Peroxide and Root Nodule Symbiosis of *Mesorhizobium huakuii*. Curr. Microbiol..

[55] Nunes J.A., Schaefer C.E., Ferreira Júnior W.G., Neri A.V., Correa G.R., Enright N.J. (2015). Soil-vegetation relationships on a banded ironstone ‘island’, Carajás Plateau, Brazilian Eastern Amazonia. An. Acad. Bras. Ciências.

[56] Thavamani P., Samkumar R.A., Satheesh V., Subashchandrabose S.R., Ramadass K., Naidu R., Venkateswarlu K., Megharaj M. (2017). Microbes from mined sites: Harnessing their potential for reclamation of derelict mine sites. Environ. Pollut..

[57] Fuentes A., Herrera H., Charles T.C., Arriagada C. (2020). Fungal and Bacterial Microbiome Associated with the Rhizosphere of Native Plants from the Atacama Desert. Microorganisms.

[58] Herrera H., Novotná A., Ortiz J., Soto J., Arriagada C. (2020). Isolation and identification of plant growth-promoting bacteria from rhizomes of *Arachnitis uniflora*, a fully mycoheterotrophic plant in southern Chile. Appl. Soil Ecol..

[59] Fowler W.M., Fontaine J.B., Enright N.J., Veber W.P. (2015). Evaluating restoration potential of transferred topsoil. Appl. Veg. Sci..

[60] Silva J.R., Gastauer M., Ramos S.J., Mitre S.K., Neto A.E.F., Siqueira J.O., Caldeira C.F. (2018). Initial growth of Fabaceae species: Combined effects of topsoil and fertilizer application for mineland revegetation. Flora.

[61] Dastogeer K.M., Li H., Sivasithamparam K., Wylie S.J. (2018). In vitro salt and thermal tolerance of fungal endophytes of *Nicotiana* spp. growing in arid regions of north-western Australia. Arch. Phytopathol. Plant Prot..

[62] Dardanelli M.S., González P.S., Medeot D.B., Paulucci N.S., Bueno M.A., Garcia M.B. (2009). Effects of peanut rhizobia on the growth and symbiotic performance of *Arachis hypogaea* under abiotic stress. Symbiosis.

[63] Zhan F., Li B., Jiang M., Li T., He Y., Li Y., Wang Y. (2019). Effects of arbuscular mycorrhizal fungi on the growth and heavy metal accumulation of bermudagrass [*Cynodon dactylon* (L.) Pers.] grown in a lead–zinc mine wasteland. Int. J. Phytoremediat..

[64] Trindade F.C., Ramos S.J., Gastauer M., Saraiva A.M.M., Caldeira C.F., Oliveira G., da Silva Valadares R.B. (2020). Metaproteomes reveal increased capacity for stress tolerance of soil microbes in ferruginous tropical rocky outcrops. Pedobiologia.

[65] Viana P.L., Mota N.F.d.O., Gil A.d.S.B., Salino A., Zappi D.C., Harley R.M., Ilkiu-Borges A.L., Secco R.d.S., Almeida T.E., Watanabe M.T.C. (2016). Flora das cangas da Serra dos Carajás, Pará, Brasil: História, área de estudos e metodologia. Rodriguésia.

[66] Kirk P.L. (1950). Kjeldahl method for total nitrogen. Anal. Chem..

[67] Kettler T., Doran J.W., Gilbert T. (2001). Simplified method for soil particle-size determination to accompany soil-quality analyses. Soil Sci. Soc. Am. J..

[68] do Nascimento S.V., Magalhaes M.M., Cunha R.L., de Oliveira Costa P.H., de Oliveira Alves R.C., de Oliveira G.C., da Silva Valadares R.B. (2018). Differential accumulation of proteins in oil palms affected by fatal yellowing disease. PLoS ONE.

[69] Herrera H., Valadares R., Oliveira G., Fuentes A., Almonacid L., do Nascimento S.V., Bashan Y., Arriagada C. (2018). Adaptation and tolerance mechanisms developed by mycorrhizal *Bipinnula fimbriata* plantlets (Orchidaceae) in a heavy metal-polluted ecosystem. Mycorrhiza.

[70] Mao X., Cai T., Olyarchuk J.G., Wei L. (2005). Automated genome annotation and pathway identification using the KEGG Orthology (KO) as a controlled vocabulary. Bioinformatics.

[71] Oliveira R.R., Silva R.L., Nunes G.L., Oliveira G. (2021). PIMBA: A PIpeline for MetaBarcoding Analysis. bioRxiv.

[72] Zhang J., Kobert K., Flouri T., Stamatakis A. (2014). PEAR: A fast and accurate Illumina Paired-End reAd mergeR. Bioinformatics.

[73] Abarenkov K., Henrik Nilsson R., Larsson K.H., Alexander I.J., Eberhardt U., Erland S., Høiland K., Kjøller R., Larsson E., Pennanen T. (2010). The UNITE database for molecular identification of fungi–recent updates and future perspectives. New Phytol..

[74] Cole J.R., Wang Q., Fish J.A., Chai B., McGarrell D.M., Sun Y., Brown C.T., Porras-Alfaro A., Kuske C.R., Tiedje J.M. (2014). Ribosomal Database Project: Data and tools for high throughput rRNA analysis. Nucleic Acids Res..

[75] Nguyen N.H., Song Z., Bates S.T., Branco S., Tedersoo L., Menke J., Schilling J.S., Kennedy P.G. (2016). FUNGuild: An open annotation tool for parsing fungal community datasets by ecological guild. Fungal Ecol..

[76] Louca S., Parfrey L.W., Doebeli M. (2016). Decoupling function and taxonomy in the global ocean microbiome. Science.

