# Supplementary material for: Proteomic Profiling and Rhizosphere-Associated Microbial Communities Reveal Adaptive Mechanisms of Dioclea apurensis Kunth in Eastern Amazon’s Rehabilitating Minelands
Source: Plants (Basel). 2022 Mar 7;11(5):712. doi: 10.3390/plants11050712 (PMC8912737; doi:10.3390/plants11050712)
Supplement: Supplementary file 1 [file plants-11-00712-s001.zip › plants-1585344-supplementary.pdf]

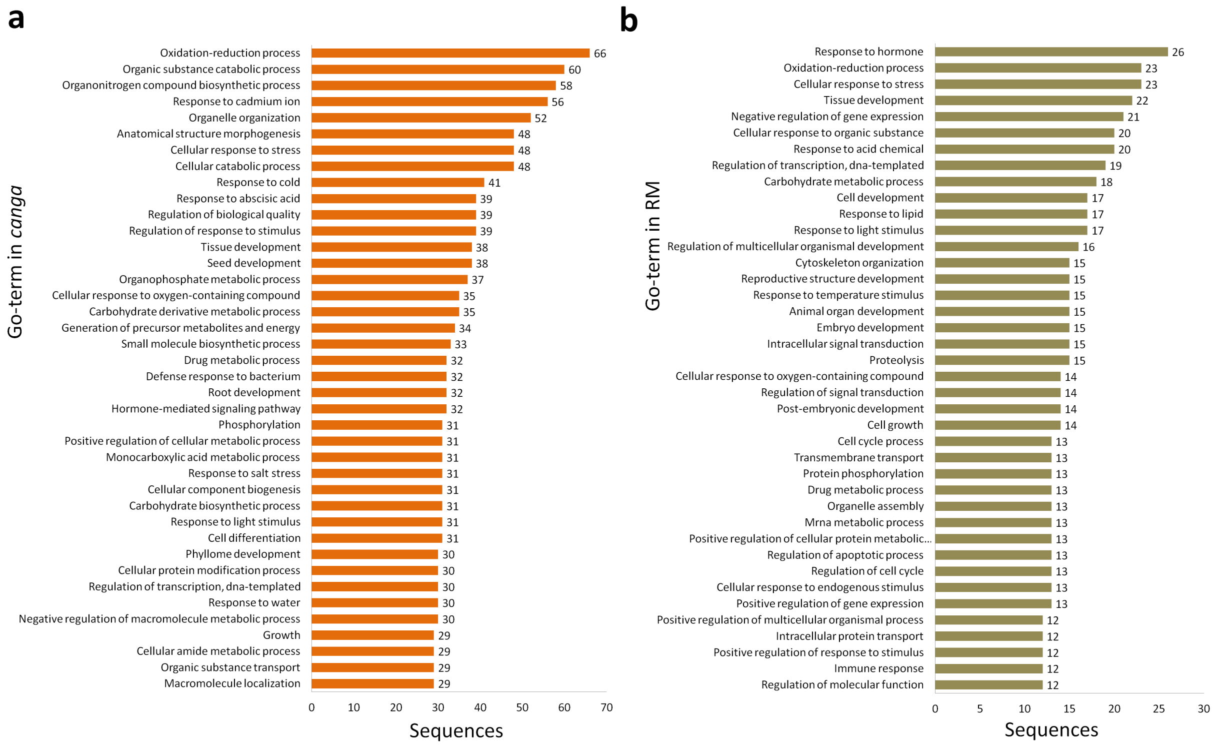

Figure S1. Top 40 categories obtained by functional annotation in different biological processes. (a) Most abundant proteins in *canga* plants and (b) the most abundant proteins in rehabilitating minelands (RM) plants.

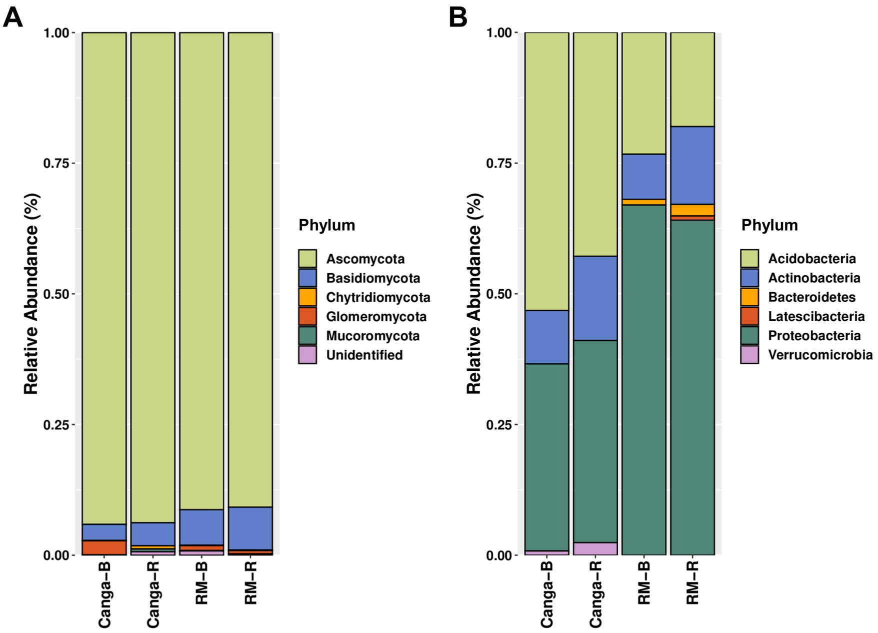

Figure S2. Relative abundance at the phylum level of major fungal (A) and bacterial (B) sequences associated with the rhizosphere (R) or bulk soil (B) of *Dioclea apurensis* growing in *canga* (*canga*) or a rehabilitating mineland (RM) in Serra dos Carajás, eastern Amazon (n = 4).

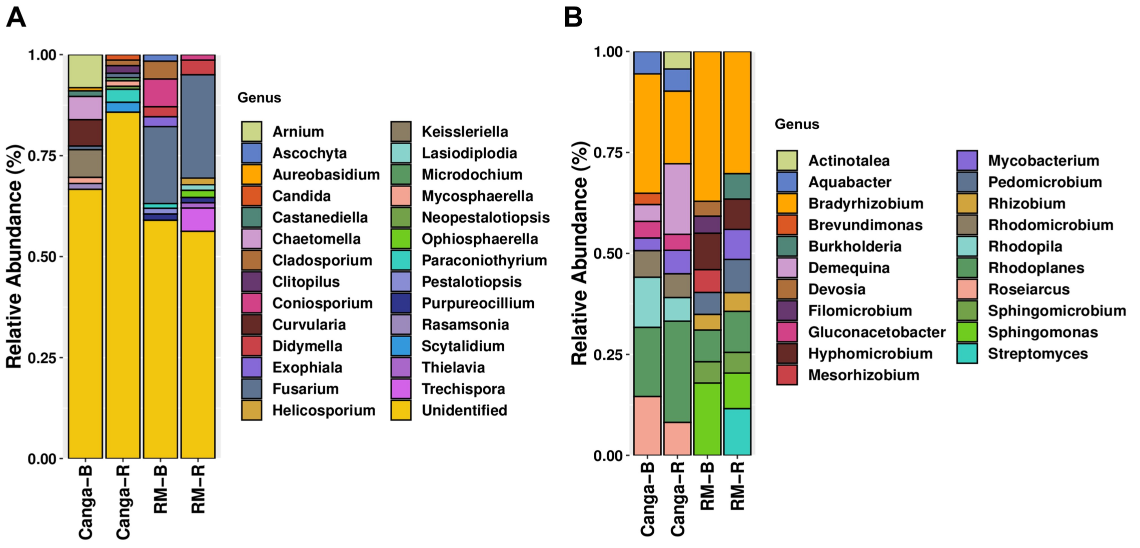

Figure S3. Relative abundance at the genus level of major fungal (A) and bacterial (B) sequences associated with the rhizosphere (R) or bulk soil (B) of *Dioclea apurensis* growing in *canga* (*canga*) or a rehabilitating mineland (RM) in Serra dos Carajás, eastern Amazon (n = 4).

Table S1. Fungal 18S rRNA sequences obtained in rhizospheric and bulk substrates samples from *Dioclea apurensis* growing in *canga* (*canga*) or rehabilitating minelands (RM).

	Sample site	Soil source	Sample ID	Total reads	Read after filtering	Number of OTU
Dioclea apurensis	*Canga*	Rhizosphere	canga_R1	210743	158066	498
			canga_R2	255066	195836	780
			canga_R3	188252	145171	494
			canga_R4	233956	192757	619
		Bulk	canga_B1	277357	208045	558
			canga_B2	231010	167332	431
			canga_B3	244945	171656	359
			canga_B4	233956	165882	430
	RM	Rhizosphere	RM_R1	205278	231358	622
			RM_R2	266446	249923	707
			RM_R3	167759	197067	789
			RM_R4	185793	161247	563
		Bulk	RM_B1	306229	169977	511
			RM_B2	310015	103815	538
			RM_B3	327132	119698	520
			RM_B4	304895	122836	591

Table S2. Bacterial 16S rRNA sequences obtained in rhizospheric and bulk substrates samples from *Dioclea apurensis* growing in *canga* (*canga*) or rehabilitating minelands (RM**).**

<table>
<tr><th></th><th>Sample site</th><th>Soil source</th><th>Sample ID</th><th>Total reads</th><th>Read after filtering</th><th>Number of OTU</th></tr>
<tr><td rowspan="16">Dioclea apurensis</td><td rowspan="8">Canga</td><td rowspan="4">Rhizosphere</td><td>canga_R1</td><td>183728</td><td>87553</td><td>559</td></tr>
<tr><td>canga_R2</td><td>155461</td><td>65746</td><td>590</td></tr>
<tr><td>canga_R3</td><td>699536</td><td>234396</td><td>632</td></tr>
<tr><td>canga_R4</td><td>295895</td><td>138631</td><td>650</td></tr>
<tr><td rowspan="4">Bulk</td><td>canga_B1</td><td>158454</td><td>9975</td><td>339</td></tr>
<tr><td>canga_B2</td><td>105497</td><td>9128</td><td>225</td></tr>
<tr><td>canga_B3</td><td>140640</td><td>18183</td><td>205</td></tr>
<tr><td>canga_B4</td><td>232601</td><td>29125</td><td>281</td></tr>
<tr><td rowspan="8">RM</td><td rowspan="4">Rhizosphere</td><td>RM_R1</td><td>577622</td><td>29185</td><td>605</td></tr>
<tr><td>RM_R2</td><td>310612</td><td>17404</td><td>477</td></tr>
<tr><td>RM_R3</td><td>427487</td><td>23526</td><td>499</td></tr>
<tr><td>RM_R4</td><td>313837</td><td>14678</td><td>441</td></tr>
<tr><td rowspan="4">Bulk</td><td>RM_B1</td><td>168299</td><td>215278</td><td>980</td></tr>
<tr><td>RM_B2</td><td>110910</td><td>242902</td><td>996</td></tr>
<tr><td>RM_B3</td><td>188221</td><td>112731</td><td>901</td></tr>
<tr><td>RM_B4</td><td>139459</td><td>113418</td><td>888</td></tr>
</table>

Table S3. Most abundant fungal and bacterial taxa identified in *Dioclea apurensis* soil substrates in plants from *canga* (*canga*) and rehabilitating minelands (RM). Numbers between parentheses are the relative abundance percentage (RA %) of each identified taxa.

**Identification Source**			**Identified Phyla (RA %)**	**Identified Genera (RA %)**
Fungi	*Canga*	*Rhizosphere*	Ascomycota (93.80); Basidiomycota (4.40); Unidentified (0.67); Chytridiomycota (0.59); Mucoromycota (0.54)	Unidentified (77.30); *Paraconiothyrium* (2.90); *Scytalidium* (2.20); *Clitopilus* (1.7); *Mycosphaerella* (1.25); *Cladosporium* (1.24); *Candida* (1.20); *Fusarium* (0.97); *Microdochium* (0.71); *Neopestalotiopsis* (0.65)
		Bulk	Ascomycota (94.10); Basidiomycota (3.10); Glomeromycota (2.69); Unidentified (0.09); Chytridiomycota (0.02)	Unidentified (61.90); *Arnium* (7.60); *Keissleriella* (6.40); *Curvularia* (6.10); *Chaetomella* (5.33); *Mycosphaerella* (1.38); *Rasamsonia* (1.35); *Castanediella* (1.29); *Fusarium* (0.80); *Aureobasidium* (0.70)
	RM	*Rhizosphere*	Ascomycota (90.80); Basidiomycota (8.20); Glomeromycota (0.66); Unidentified (0.25); Chytridiomycota (0.09)	Unidentified (49.90); *Fusarium* (22.70); *Trechispora* (5.10); *Didymella* (3.20); *Ophiosphaerella* (1.58); *Helicosporium* (1.45); *Lasiodiplodia* (1.22); *Purpureocillium* (1.17); *Coniosporium* (1.13); *Thielavia* (1.2)
		Bulk	Ascomycota (91.30); Basidiomycota (6.82); Glomeromycota (1.0); Unidentified (0.83); Mucoromycota (0.04); Chytridiomycota (0.01)	Unidentified (52.40); *Fusarium* (16.90); *Coniosporium* (6.00); *Cladosporium* (3.92); *Didymella* (2.21); *Exophiala* (2.17); *Ascochyta* (1.41); *Purpureocillium* (1.36); *Pestalotiopsis* (1.27); *Paraconiothyrium* (1.02)
Bacteria	*Canga*	*Rhizosphere*	Acidobacteria (42.80); Proteobacteria (38.70); Actinobacteria (16.10); Verrucomicrobia (2.40)	*Rhodoplanes* (20.00); *Bradyrhizobium* (14.40); *Demequina* (13.90); *Roseiarcus* (6.54); *Rhodomicrobium* (4.74); *Mycobacterium* (4.67); *Rhodopila* (4.66); *Aquabacter* (4.38); *Actinotalea* (3.47); *Gluconacetobacter* (3.17)
		Bulk	Acidobacteria (53.20); Proteobacteria (35.80); Actinobacteria (10.20); Verrucomicrobia (0.80)	*Bradyrhizobium* (24.30); *Rhodoplanes* (14.00); *Roseiarcus* (11.90); *Rhodopila* (10.20); *Rhodomicrobium* (5.42); *Aquabacter* (4.57); *Demequina* (3.44); *Gluconacetobacter* (3.42); *Mycobacterium* (2.54); *Brevundimonas* (2.29)
	RM	*Rhizosphere*	*Proteobacteria* (64.10); *Acidobacteria* (18.00); *Actinobacteria* (14.90); *Bacteroidetes* (2.20); *Latescibacteria* (0.80)	*Bradyrhizobium* (14.50); *Streptomyces* (5.50); *Rhodoplanes* (4.80); *Sphingomonas* (4.20); *Pedomicrobium* (3.95); *Hyphomicrobium* (3.61); *Mycobacterium* (3.55); *Burkholderia* (3.01); *Sphingomicrobium* (2.44); *Rhizobium* (2.30)
		Bulk	Proteobacteria (67.00); Acidobacteria (23.30); Actinobacteria (8.63); Bacteroidetes (1.07)	*Bradyrhizobium* (18.50); *Sphingomonas* (12.00); *Hyphomicrobium* (6.00); *Rhodoplanes* (5.30); *Mesorhizobium* (3.81); *Pedomicrobium* (3.68); *Sphingomicrobium* (3.55); *Filomicrobium* (2.86); *Rhizobium* (2.61); *Devosia* (2.48)

Table S4. Steps and procedures of protein extraction**.**

Steps	Procedure
**1**	The roots of five plants were mixed for each condition and the material was macerated in liquid nitrogen. Were using about 300 mg of macerated roots.
**2**	In each sample were added 10 mL of buffer containing sucrose (1.5 M), Tris-Hydrochloride (1M, pH 8), sodium dodecyl sulfate (SDS, 10%), Phenylmethylsulfonyl fluoride (PMSF, 100 mM), polyvinylpolypyrrolidone (PVPP), ultrapure water with addition of 100 µL of protease inhibitor (SIGMA) and 500 µL of β-mercaptoethanol.
**3**	The samples were sonicated for five repetitions of 30 seconds at room temperature.
**4**	The extracts were divided in ten microtubules each, followed by the addition of 700 µL of phenol per microtubule. The samples were vortexed by 15 minutes and centrifuged during 8 minutes at 14000 rpm, for the phenolic phase separation.
**5**	The phenolic content was transferred to new microtubule and the operation repeated to eliminate the any residue of aqueous phase or SDS.
**6**	There were added 1300 µL of ammonia acetate (100 mM) in methanol (100%) for protein precipitation during 24 hours at -80 ºC.
**7**	Centrifugation at 14000 rpm during 8 minutes, and the supernatant was discarded. The precipitate was transferred to a new microtubule and washed four times with acetone 80%.
**8**	The last washing was made with ethanol 70% and the precipitate dried at room temperature in vacuum concentrator around seven minutes.
**9**	The extracts were solubilized in 200 µL of RapiGest (Waters, Milford, MA, USA) 0,2%.
